# Supplementary material for: Electronic inhomogeneity and phase fluctuation in one-unit-cell FeSe films
Source: Nat Commun. 2024 Apr 20;15:3369. doi: 10.1038/s41467-024-47350-0 (PMC11032316; doi:10.1038/s41467-024-47350-0)
Supplement: Supplementary file 1 — Supplementary Information [file 41467_2024_47350_MOESM1_ESM.pdf]

Electronic inhomogeneity and phase fluctuation in one-unit-cell FeSe films

Supplementary Information

Dapeng Zhao[1†], Wenqiang Cui[1,2†], Yaowu Liu[2†], Guanming Gong[2], Liguo Zhang[1], Guihao Jia[2], Yunyi Zang[1], Xiaopeng Hu[2], Ding Zhang[1,2,3], Yilin Wang[4*], Wei Li[2,3], Shuaihua Ji[2,3], Lili Wang[2,3*], Ke He[1,2,3], Xucun Ma[2,3], and Qi-Kun Xue[1,2,3,5*]

[1]Beijing Academy of Quantum Information Sciences, Beijing 100193, China
[2]State Key Laboratory of Low-Dimensional Quantum Physics, Department of Physics, Tsinghua University, Beijing 100084, China
[3]Frontier Science Center for Quantum Information, Beijing 100084, China
[4]School of Integrated Circuits, Shandong Technology Center of Nanodevices and Integration, State Key Laboratory of Crystal Materials, Shandong University, Jinan 250100, China
[5]Department of Physics, Southern University of Science and Technology, Shenzhen 518055, China
[†]These authors contributed equally to this work. [*]e-mail: qkxue@mail.tsinghua.edu.cn; liliwang@mail.tsinghua.edu.cn; yilinwang@email.sdu.edu.cn

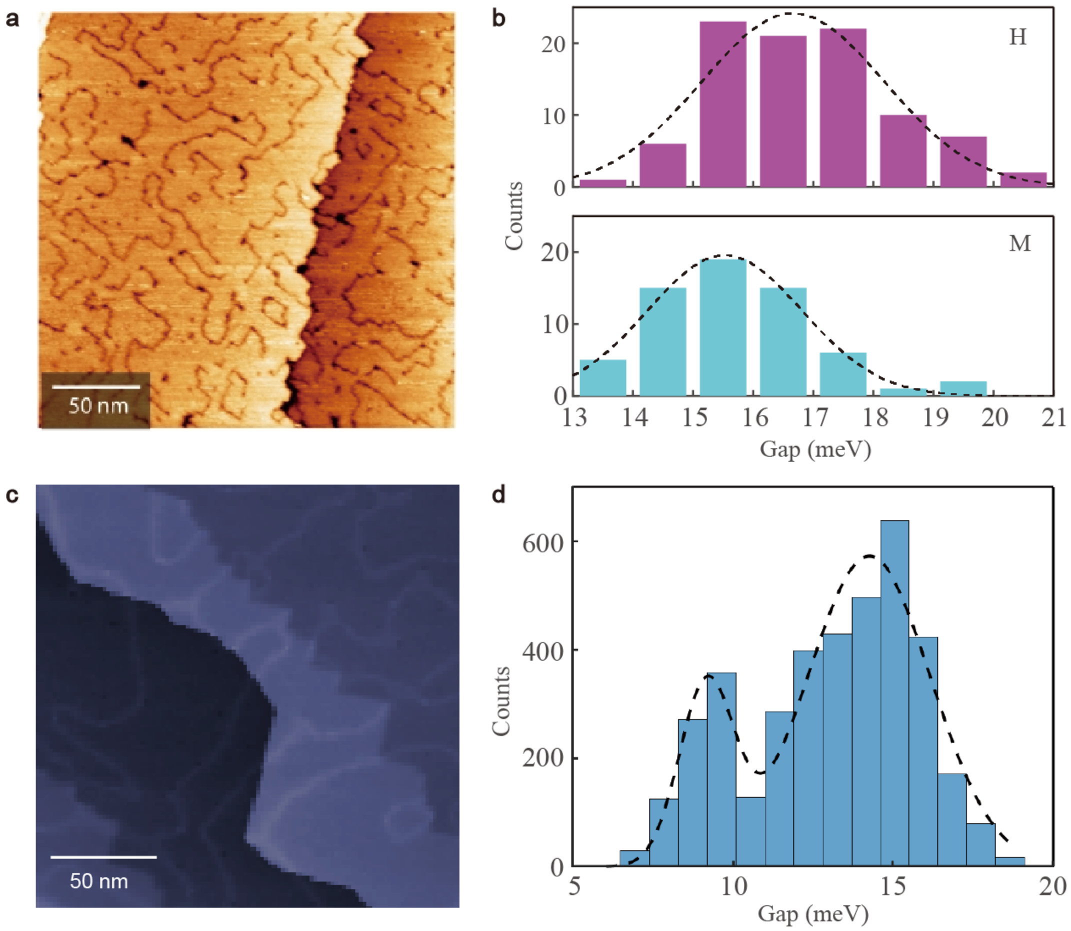

Fig. S1 | The STM morphology and statistics on gap magnitude for 1 UC FeSe film with **a**-**b** (extracted form literatures 1,2), line defects and **c**-**d**, bright twin boundaries.

The 1 UC FeSe films on $SrTiO_3$(001) consist of two types of domain boundaries, i.e. line defects under low Se flux with strong interface coupling and apparent bright twin boundaries under high Se flux with weakened interface coupling. As shown in Fig. S1a, under strong interface coupling limit, the domains in 1 UC FeSe film are separated by unidirectional line defects of Fe vacancies, wherein the outer gap reaches at 20 meV in maximum. Around line defects, the superconductivity gets suppressed[3]. The gap magnitude also decreases with reduced domain size. Thus, 1 UC FeSe films with line defects boundaries show a nearly Gaussian-distributed spatial gap distribution[2], regardless of the value of gap magnitude, as shown in Fig. S1b. The different statistical results in locations H and M demonstrate the inhomogeneity in millimeter-scale. Whereas, under weakened interface coupling, the domains in 1 UC FeSe film are separated by bright twin boundaries, which are also FeSe films with compressed lattice, as shown in Fig. S1c. Without the dense line defects, 1 UC FeSe films with twin boundaries exhibit two typical superconducting gaps, namely ~15 meV in the domain and ~10 meV on twin boundaries. The representative gap distribution, as shown in Fig. S1d, is extracted from the sample in this study, which is also consistent with a previous study[4].

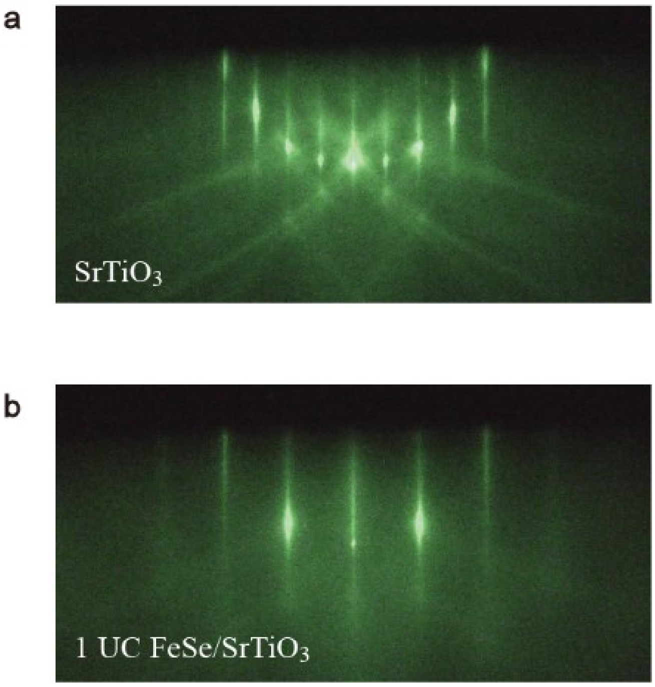

Fig. S2 | Reflection high-energy electron diffraction (RHEED) patterns of **a**, Nb doped conductive $SrTiO_3$ substrate and **b**, 1 UC FeSe film on $SrTiO_3$.

Sharp streaky patterns indicate the good crystalline quality of $SrTiO_3$ substrates and FeSe films. After degassing at 600℃ for hours and annealing at 1200℃ for 20 min in the MBE chamber, the $SrTiO_3$ substrate shows 2×2 reconstructions, which is confirmed by STM.

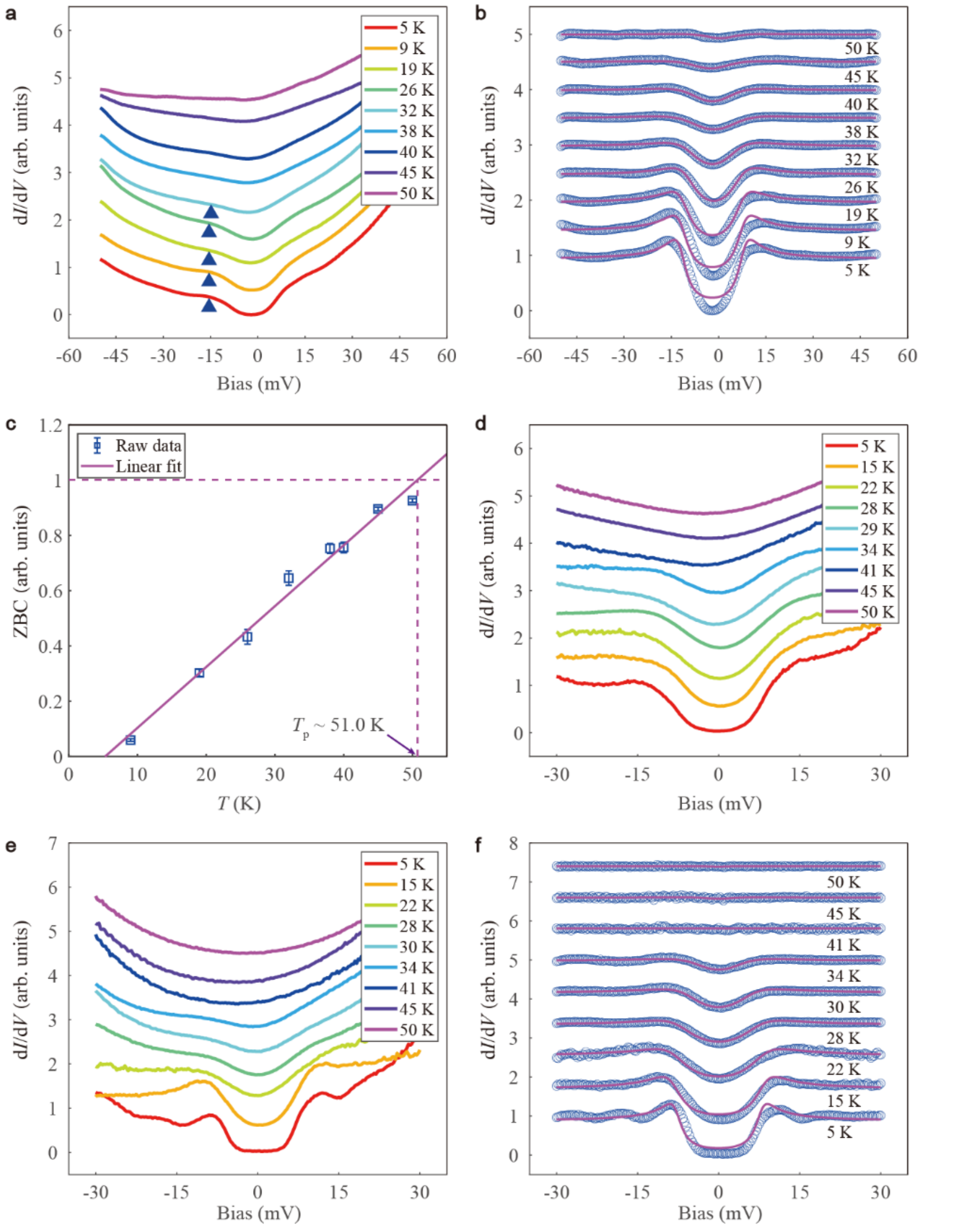

Fig. S3 | Fitting results of d*I*/d*V* spectra. a, The original d*I*/d*V* spectra taken in domain at different temperatures. The triangles mark the coherence peak. Above 32 K, no obvious kink can be observed. **b**, Normalized spectra (open symbols) and BCS fittings (solid curves) at each temperature. **c**, The temperature dependence of the zero bias conductance (ZBC) extracted from the d*I*/d*V* spectra in **b**. The error bars are from the s.d. of the fittings. **d**, another dataset of original d*I*/d*V* spectra taken in domain. **e**, The original d*I*/d*V* spectra taken on twin boundaries. **f**, Normalized spectra (open symbols) and BCS fittings (solid curves) at each temperature.

Table S1 | Dynes-fitting parameters [$\Delta(T)$ and $\Gamma(T)$] for fitting the spectra in Fig. S3b

T (K)	Δ (meV)	Γ (meV)
5	10.02	2.51
9	9.80	2.79
19	8.64	3.30
26	8.37	4.21
32	7.61	5.60
38	7.00	7.29
40	6.54	7.02
45	4.86	6.92
50	3.74	7.00

Table S2 | Dynes-fitting parameters [$\Delta(T)$ and $\Gamma(T)$] for fitting the spectra in Fig. S3f

T (K)	Δ (meV)	Γ (meV)
5	7.79	1.59
15	8.61	2.44
22	8.05	3.98
28	7.17	4.51
30	5.84	4.60
34	4.08	4.85
41	0	/
45	0	/
50	0	/

The BCS fitting of dI/dV spectra was conducted using Dynes function[5]. Each spectrum is first normalized by dividing a polynomial background fit and then fitted using Dynes function. Figure S3a,b represent the raw dI/dV spectra in domain and the fitting results, respectively. Besides, the ZBC shows a linear temperature dependence, as shown in Fig. S3c, giving a $T_p \sim 51$ K when ZBC=1, which is consistent with the gap closing temperature. Figure S3d shows another dataset of the original dI/dV spectra, demonstrating the repeatability and reliability of the results. Figure S3e,f represent the raw dI/dV spectra on twin boundaries and the fitting results, respectively. The spectral broadening Γ is a parameter in the Dynes fitting, which is related with the quasiparticle lifetime. Tables S1 and S2 show Γ used for fitting the spectra in Figs. S3b and S3f at different temperatures, respectively.

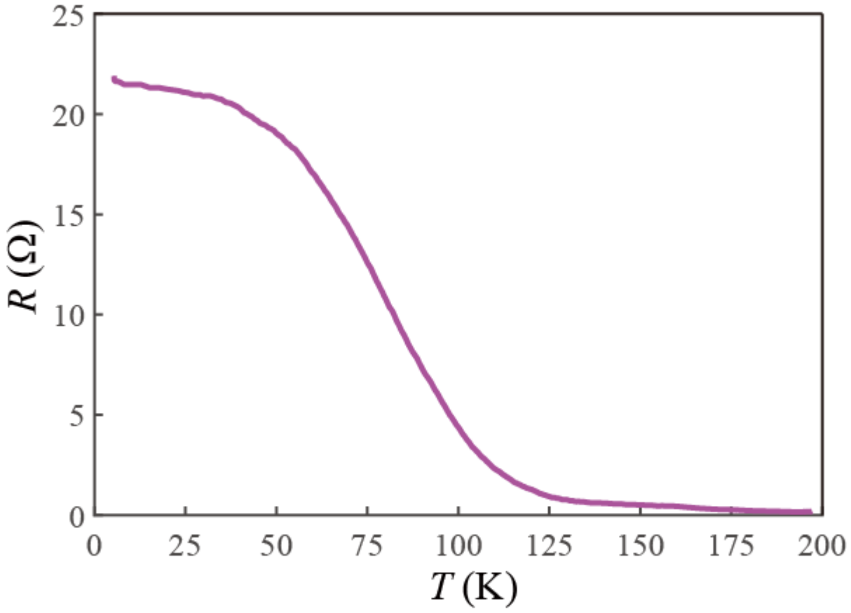

Fig. S4 | Temperature-dependent resistance of Nb-doped $SrTiO_3$ substrate.

Figure S4 indicates that the Nb-doped $SrTiO_3$ substrate has no influence on the measurements of superconducting transition in 1 UC FeSe films at low temperature. Au electrodes were deposited on the $SrTiO_3$ substrate before conducting the measurements, because the electrical connection between the micro-four-point probes and the substrate can hardly be reached by direct contact. Due to the spread of Au atoms while passing through the mask, the actual measured distance is much shorter than 5 μm.

Table S3 | Comparison of representative T_c measurements on FeSe/$SrTiO_3$(001) across different techniques

Heterostructure	T_c	Technique	Reference
1 UC FeSe/Nb:$SrTiO_3$	T_c > 42.9 K	STM/STS	6
1 UC FeSe/$SrTiO_3$	T_c ~ 68 K	STM/STS	7
1 UC FeSe/Nb:$SrTiO_3$	T_c ~ 65±5 K	ARPES	8
1 UC FeSe/Nb:$SrTiO_3$	T_c ~ 60±5 K	ARPES	9
1 UC FeSe/Nb:$SrTiO_3$	T_c ~ 58±7 K	ARPES	10
1 UC FeSe/$SrTiO_3$	T_c ~ 50-60 K	ARPES	11
1 UC FeSe/Nb:$SrTiO_3$	T_c ~ 83 K	ARPES	12
1 UC FeSe/$SrTiO_3$	T_c ~ 73±5 K	ARPES	13
Si/5 UC FeSe/$SrTiO_3$	T_c^{zero} < 30 K, T_c^{onset} ~ 53 K	*Ex-situ* transport	6
Si/10 UC FeTe/1 UC FeSe/$SrTiO_3$	T_c^{zero} ~ 23.5 K, T_c^{onset} ~ 40.2 K	*Ex-situ* transport	14
1 UC FeSe/Nb:$SrTiO_3$	T_c^{zero} ~ 109 K	*In-situ* transport	15
1 UC FeSe/$SrTiO_3$	T_c^{zero} ~ 13 K, T_c^{onset} ~ 40 K	*In-situ* transport	16
1 UC FeSe/$SrTiO_3$	T_c^{zero} ~ 29±0.2 K, T_c^{onset} ~ 44±3 K	*In-situ* transport	13
Si/10 UC FeTe/1 UC FeSe/$SrTiO_3$	T_c ~ 21 K	Magnetization	14
Si/10 UC FeTe/3-4 UC FeSe/$SrTiO_3$	T_c ~ 20-45 K	Magnetization	17
10 UC FeTe/1 UC FeSe/Nb:$SrTiO_3$	T_c ~ 85 K	Magnetization	18

There are a lot of *ex-situ* and *in-situ* measurements on 1 UC FeSe/$SrTiO_3$ in recent years, however, the consensus on its T_c has not been reached for the time being. Table S3 presents discrepancies on T_c even with the same measurement technique, which is probably due to different sample quality, such as preparation conditions, capping layers and types of domain boundaries. Reference 8 shows that different annealing conditions give different superconducting gaps, and larger gaps usually have higher closing temperatures. This feature of 1 UC FeSe demonstrates the importance of combining STM/STS and *in-situ* microscale transport measurements, enabling to know the sample status down to atomic scale. Overall, previous studies suggest the most common results that the gap closing temperature is ~65 K, T_c^{zero} ranges from 20 K to 29 K and T_c^{onset} ranges from 40 K to 53 K. Compared with the gap closing temperature ~ 52 K in this study, 65 K is likely obtained on FeSe films with strong interface coupling, featured by line defects domain boundaries and larger superconducting gaps. Besides, T_c^{zero} in this study is higher than 30 K, demonstrating the good sample quality and improved homogeneity under micro-scale measurements. Our result indicates that T_c^{onset} is consistent with the pairing temperature. Despite the different definition of T_c^{onset}, the relatively low T_c^{onset} in previous studies may be caused by the influence of capping layers or inhomogeneity[19,20].

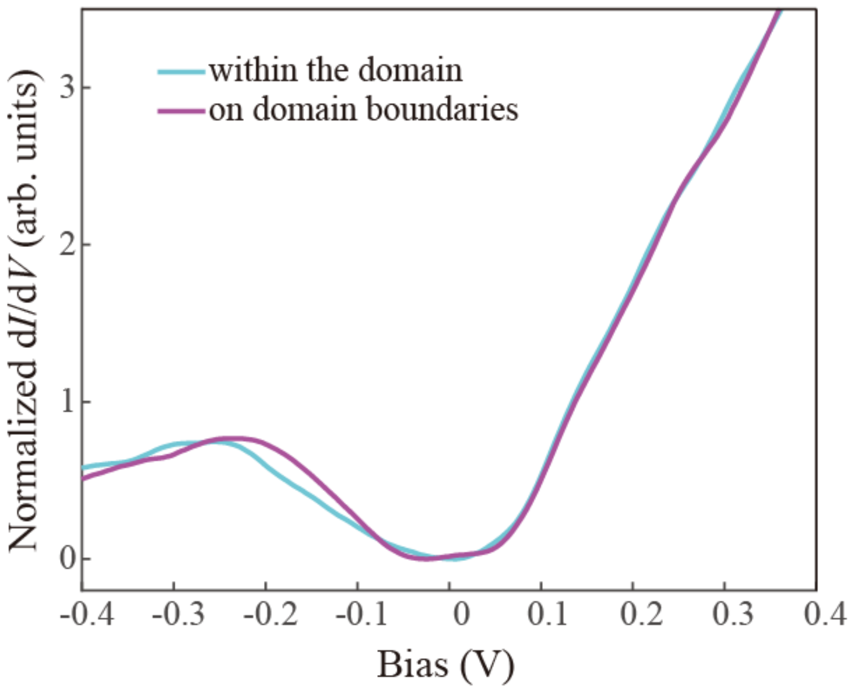

Fig. S5 | Comparison of large-energy-scale dI/dV spectra (V_S = 0.4 V, I_t = 100 pA) of FeSe films within the domain and on twin boundaries.

Figure S5 shows a blue-shifted kink of valence band on twin boundaries, compared with FeSe films within the domain.

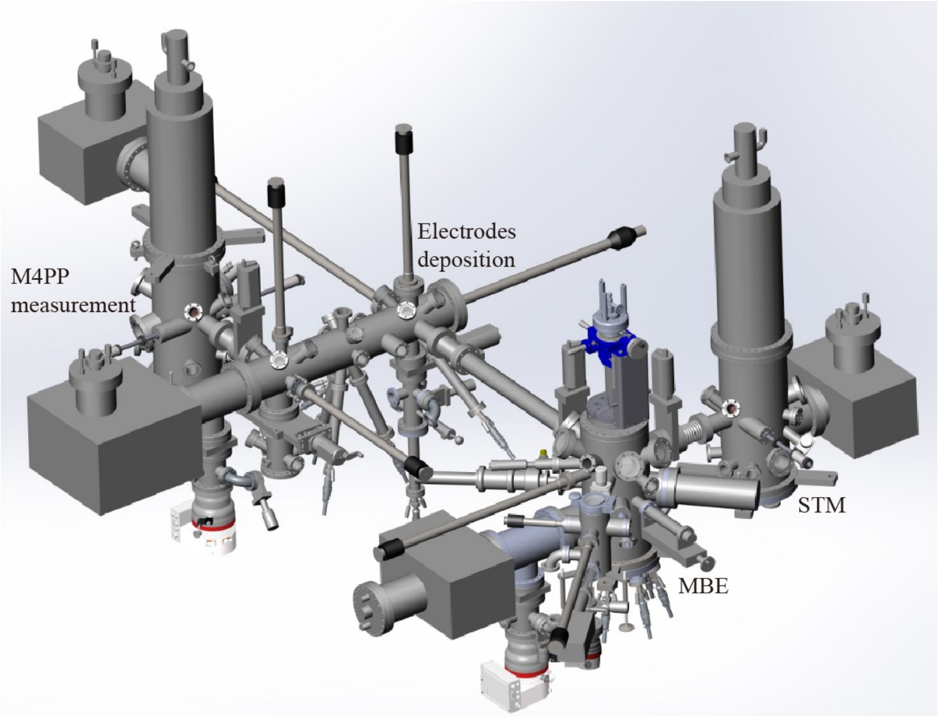

Fig. S6 | Schematic of the combined MBE - STM - *in-situ* micron-scale electrical transport measurement system.

This system allows MBE growth, STM characterization, electrode deposition and micro-four-point probes (M4PP) electrical transport measurement to be performed under the same ultra-high vacuum. Our experiments were conducted in this system.

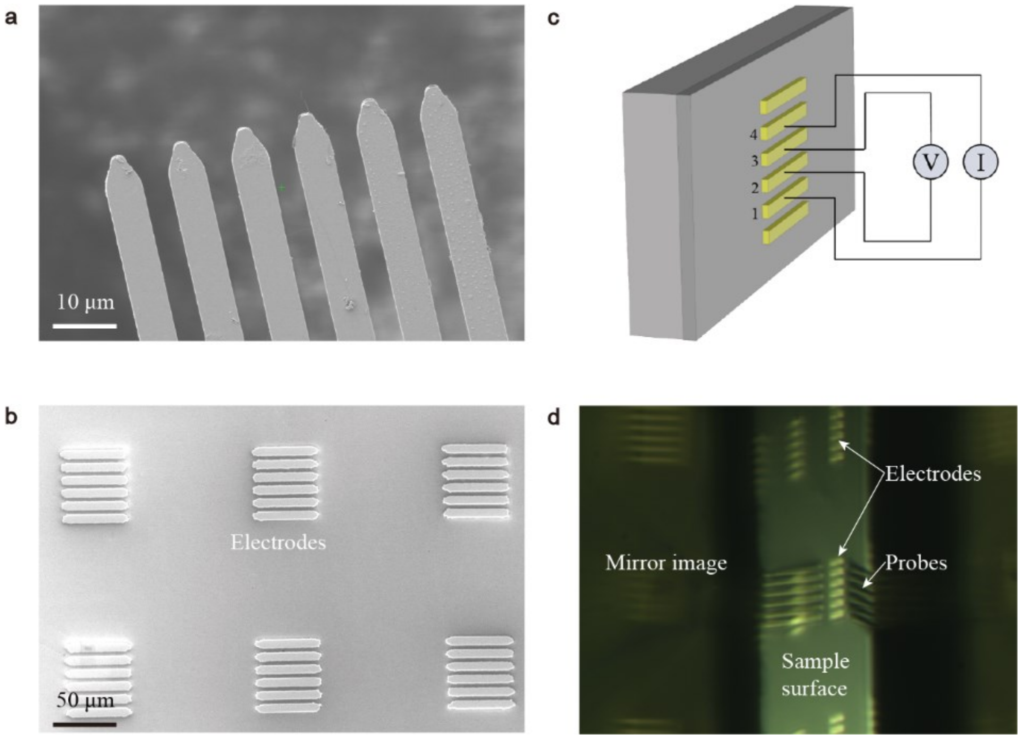

Fig. S7 | Illustration of *in-situ* micron-scale electrical transport measurements. a, Scanning electron microscopy (SEM) image of the M4PP. **b**, SEM image of Au electrodes. **c**, Electrical circuit for transport measurement. **d**, Optical image of the measurement process.

Figures S7a,b show the SEM images of the M4PP and Au electrodes, respectively. Each M4PP chip consists of six probes, where four probes are used for transport measurement and two probes are for backup. The width of each probe is 5 μm. The distance between adjacent probes is also 5 μm. The electrodes match well with the M4PP. Figure S7c shows the electrical circuit for transport measurement. Four probes are electrically connected to a Keithley 6221 DC Sourcemeter and a Keithley 2182A Nanovoltmeter, respectively. Based on the DC current-reversal technique, a built-in Pulse Delta measurement mode of the nanovoltmeter is used to cancel the effects of thermoelectric potentials, which are generated by thermal differences between the junctions of probes and the sample. Specifically, the 2182A Nanovoltmeter performs the first voltage measurement (V_1) while sourcing a pulsed DC current (I), and performs the second voltage measurement (V_2) while sourcing a reversed current ($-I$). Then the final voltage is calculated as $(V_1 - V_2)/2$. Moreover, the pulsed current could avoid heating effects during measurements. Figure S7d shows the optical image of the measurement process. Monitored by the optical microscope, we can locate the measurement area at the micro scale. Note that transport measurements in this paper were conducted directly on the sample surface, as shown in Fig. 2, even though electrodes could facilitate keeping good contacts while varying temperature. This is because electrodes suppress the superconductivity of 1 UC FeSe films.

References

1. Zhang, W. et al. Interface charge doping effects on superconductivity of single-unit-cell FeSe films on $SrTiO_3$ substrates. *Phys. Rev. B* **89**, 060506 (2014).
2. Gong, G. et al. Oxygen vacancy modulated superconductivity in monolayer FeSe on $SrTiO_{3-\delta}$. *Phys. Rev. B* **100**, 224504 (2019).
3. Jiao, X. et al. Post-growth Fe deposition on the superconductivity of monolayer FeSe films on $SrTiO_{3-\delta}$. *Phys. Rev. Mater.* **6**, 064803 (2022).
4. Fan, Q. et al. Plain s-wave superconductivity in single-layer FeSe on $SrTiO_3$ probed by scanning tunnelling microscopy. *Nat. Phys.* **11**, 946-953 (2015).
5. Dynes, R. C. et al. Direct measurement of quasiparticle-lifetime broadening in a strong-coupled superconductor. Phys. Rev. Lett. **41**, 1509 (1978).
6. Wang, Q.-Y. et al. Interface-induced high-temperature superconductivity in single unit-cell FeSe films on $SrTiO_3$. *Chin. Phys. Lett.* **29**, 037402 (2012).
7. Zhang, W. et al. Interface charge doping effects on superconductivity of single-unit-cell FeSe films on $SrTiO_3$ substrates. *Phys. Rev. B* **89**, 060506 (2014).
8. He, S. et al. Phase diagram and electronic indication of high-temperature superconductivity at 65 K in single-layer FeSe films. *Nat. Mater.* **12**, 605-610 (2013).
9. Tan, S. et al. Interface-induced superconductivity and strain-dependent spin density waves in FeSe/$SrTiO_3$ thin films. *Nat. Mater.* **12**, 634-640 (2013).
10. Lee, J. J. et al. Interfacial mode coupling as the origin of the enhancement of T_c in FeSe films on $SrTiO_3$. *Nature* **515**, 245-248 (2014).
11. Yang, M. et al. Light induced non-volatile switching of superconductivity in single layer FeSe on $SrTiO_3$ substrate. *Nat. Commun.* **10**, 85 (2019).
12. Xu, Y. et al. Spectroscopic evidence of superconductivity pairing at 83 K in single-layer FeSe/$SrTiO_3$ films. *Nat. Commun.* **12**, 2840 (2021).
13. Faeth, B. D. et al. Incoherent cooper pairing and pseudogap behavior in single-layer FeSe/$SrTiO_3$. *Phys. Rev. X* **11**, 021054 (2021).
14. Zhang, W.-H. et al. Direct observation of high-temperature superconductivity in one-unit-cell FeSe films. *Chin. Phys. Lett.* **31**, 017401 (2014).
15. Ge, J.-F. et al. Superconductivity above 100 K in single-layer FeSe films on doped $SrTiO_3$. *Nat. Mater.* **14**, 285-289 (2015).
16. Pedersen, A. K. et al. Interfacial superconductivity in FeSe ultrathin films on $SrTiO_3$ probed by *in situ* independently driven four-point-probe measurements. *Phys. Rev. Lett.* **124**, 227002 (2020).
17. Deng, L. Z. et al. Meissner and mesoscopic superconducting states in 1-4 unit-cell FeSe films. *Phys. Rev. B* **90**, 214513 (2014).
18. Sun, Y. et al. High temperature superconducting FeSe films on $SrTiO_3$ substrates. *Sci. Rep.* **4**, 6040 (2014).
19. Li, Y. et al. Capping layer influence and isotropic in-plane upper critical field of the superconductivity at the FeSe/$SrTiO_3$ interface. *Phys. Rev. Mater.* **5**, 034802 (2021).
20. Liu, Y. et al. Spatial inhomogeneity of superconducting gap in epitaxial monolayer $FeTe_{1-x}Se_x$ films. *Phys. Rev. B* **108**, 214514 (2023).
